# Using *Azolla* (*Azolla microphylla*) leaf meal and phytonutrient powder on rumen fermentation efficiency and nutrient degradability using *in vitro* technique

**DOI:** 10.5713/ab.24.0379

**Published:** 2024-10-24

**Authors:** Burarat Phesatcha, Kampanat Phesatcha, Thiwakorn Ampapon, Metha Wanapat

**Affiliations:** 1 Department of Applied Biology, Faculty of Science and Liberal Arts, Rajamangala University of Technology Isan, Nakhon Ratchasima 30000, Thailand; 2 Department of Animal Science, Faculty of Agriculture and Technology, Nakhon Phanom University, Nakhon Phanom 48000, Thailand; 3 Department of Animal Science, Faculty of Agriculture and Technology, Rajamangala University of Technology Isan, Surin 32000, Thailand; 4 Tropical Feed Resources Research and Development Center (TROFREC), Department of Animal Science, Faculty of Agriculture, Khon Kaen University, Khon Kaen 40002, Thailand

**Keywords:** *Azolla microphylla*, Degradability, Phytonutrient, Ruminal Fermentation, Turmeric Powder

## Abstract

**Objective:**

This work was to investigate the effect of using *Azolla* (*Azolla microphylla*) leaf meal and phytonutrient powder on rumen fermentation efficiency and nutrient degradability using *in vitro* technique.

**Methods:**

All respective treatments were imposed in a 2×4×2 Factorial arrangements according to a completely randomized design. The first factor was two ratios of roughage to concentrate (R:C at 60:40, and 40:60), the second factor was *Azolla* (*Azolla microphylla*) powder (AMP) supplementation levels (0%, 3%, 6%, and 9% of the total substrate) and the third factor was turmeric (*Curcuma longa*) powder (TUP) supplementation levels (0% and 2% of the total substrate).

**Results:**

Cumulative gas production at 96 h, was affected by R:C and numerically increased by AMP and TUP supplementation (p<0.05). Gas production kinetics increased with the increasing ratio of concentrate and AMP supplementation whereas TUP supplement reduced gas production. *In vitro* dry matter degradability was remarkably increased (p<0.05) by the R:C ratio, AMP and TUP supplementation. However, increasing R:C ratio, AMP and TUP supplementation resulted in the concentration of propionate (C_3_) significantly increasing (p<0.05). Acetate (C_2_), C_2_:C_3_ ratio, and protozoal population were improved (p<0.05), while the methane production decreased.

**Conclusion:**

Under this study, the results were obtained under the supplementation level of 9% AMP and 2% TUP of total substrate, hence, the combined use is potentially beneficial. These results revealed a potential use of AMP and TUP as a supplement to improve rumen fermentation for ruminant feeding. Nevertheless, in vivo feeding trials should be further investigated using AMP and TUP as a source of protein and phytonutrient.

## INTRODUCTION

One factor that contributes to increasing average global temperatures and other climatic changes is the release of greenhouse gases into the atmosphere. Manure management and animal enteric fermentation are the main sources of agricultural methane gas emissions, while manure management is the primary source of nitrous oxide emissions. The formation of enteric methane in ruminants’ rumens is heavily linked to the loss of digestible energy, which can reach as high as 12% of GE intake. Rumen fermentation efficiency can be improved if hydrogen is captured for the synthesis of propionic acid (C_3_) while methane production is minimized [1]. Some strategies have attempted to reduce methane emissions by adjusting ruminal fermentation utilizing many rumen modifying procedures. One strategy involves the utilization of plant extracts rich in phytonutrients and secondary metabolites [2]. How plant secondary metabolites impact ruminant digestion has been assessed by several *in vitro* and *in vivo* investigations using various plant extracts and essential oils [3]. Tropical plants have abundant phytonutrient components including condensed tannins (CT) and saponins. These phytonutrient components demonstrate antibacterial effects, particularly against populations of protozoa and methanogens. *Azolla sp*. has long been a staple Asian diet of pigs and poultry and as a green manure fertilizer for rice fields and agricultural development [4,5]. At least eight species of *Azolla sp*. have been identified including *Azolla mexicana*, *Azolla japonica*, *Azolla nilotica*, *Azolla caroliniana*, *Azolla circinata*, *Azolla pinnata*, *Azolla microphylla*, and *Azolla rubra* [6]. *Azolla microphylla* is a fern that floats on the surface of the water [7]. This plant has many spores, small, overlapping leaves, a bright green color, and a smooth velvety texture. Chemical analysis of the nutrients in *Azolla microphylla* revealed high protein at 31.3%, fat at 7.5%, and soluble sugar and crude fiber at 3.5% and 6%, respectively [7]. Additionally, Basak et al [8] reported that *Azolla* meal contained 25.8% crude protein (CP), 3.5% ether extract, 15.8% ash, 15.7% crude fiber, and 30.1% nitrogen-free extract after air drying. As well as its high protein, vitamin, and mineral content, *Azolla* also contains bioactive secondary metabolites like steroids, anthraquinone glycosides, sucrose, tannins, and phenols [9]. Abraham et al [10] investigated the antibacterial potential of an *A. microphylla* extract against plant pathogenic bacteria.

*Curcuma longa*, also known as turmeric, is the rhizome of an herbaceous perennial plant and a member of the Zingiberaceae family [11]. Polyphenolic compounds found in turmeric include curcuminoids which contain 70% to 77% curcumin, 18% to 20% demethoxycurcumin, and 7% to 10% bisdemethoxycurcumin [12]. Turmeric has active ingredients that exhibit a wide range of antibacterial and antifungal biological activities and is also used in traditional medicine. Turmeric is one of the many species of plants that have been utilized as raw materials for both food and non-food applications [13]. Tyagi et al [14] reported extremely potent antibacterial action by curcumin against both gram-positive and gram-negative bacteria and these plants serve as a suppressor of methane (CH_4_) production. However, limited information exists on the effect of combined *Azolla microphylla* and turmeric powder supplementation as a feed additive on ruminal fermentation efficiency and nutrient degradability. Therefore, this research study evaluated the effect of *Azolla* (*Azolla microphylla*) leaf meal and phytonutrients from turmeric powder on *in vitro* fermentation end-products, gas production, nutrient degradability, and methane mitigation using an *in vitro* gas production technique.

## MATERIALS AND METHODS

### Ethical procedure

This study was approved by the Animal Care and Use Committee of Rajamangala University of Technology Isan, Thailand (approval no. 01-66-006). According to Thailand’s National Research Council’s Ethics of Animal Experimentation, approval was required to collect rumen fluid from animals for this study’s main objective, which comprised laboratory examination of ruminant feeds.

### Dietary treatments and experimental design

Fresh *Azolla* (*Azolla microphylla*) was collected at 15 days of age and fresh turmeric rhizomes were harvested from Roi-et Province, Thailand. Fresh *Azolla* and turmeric were dried at 60°C, and then ground to pass through a 1-mm screen using a Cyclotech Mill (Hoganas, Sweden). The dietary treatments followed a 2 × 4 × 2 Factorial arrangement in a completely randomized design (CRD), with three replicates per treatment including blank triplicates (medium only). The first factor was two ratios of R:C at 60:40 and 40:60, the second factor was four supplementation levels of *Azolla* (*Azolla microphylla*) powder (AMP) at 0%, 3%, 6%, and 9% dry matter (DM) substrate, and the third factor was two supplementation levels of turmeric (*Curcuma longa*) powder (TUP) at 0 and 2% DM substrate. Details of the nutritive values of concentrate, roughage source, AMP, and TUP of the experiment are shown in Table 1. Roughage, concentrate, AMP, and TUP were dried at 60°C and passed through a screen (1 mm) to determine the chemical analysis of DM, organic matter, and CP using standard methods [15]. Following Van Soest et al [16], measurements were taken to determine the fiber content, especially neutral detergent fiber (NDF) and acid detergent fiber (ADF). Using the Folin-Ciocalteu reagent and measuring absorbance at 765 nm, the TUP was evaluated for total phenolic compounds [17], total flavonoid compounds [18], and tannin content [19].

### Rumen inoculum preparation

This experiment utilized rumen fluid as a source of fermentation from two Thai native beef cattle (2 years old) with an average live weight of 250±10 kg. Before the morning feeding, a combined volume of approximately 1,000 mL of rumen fluid was obtained from each animal. All the beef cattle were fed with rice straw ad libitum and concentrate mixture at 0.5% of body weight at 14% CP and 75% TDN. The *in vitro* fermentation technique followed Menke et al [20]. A suction pump was used to collect 1,000 mL of rumen fluid from each specimen. A feed sample from the experiment was weighed and put into 50 mL bottles of the total mixed substrate (200 mg). Before mixing with substrates in the treatments, the rumen fluid was mixed and maintained in an anaerobic condition. Bottles with the mixtures of substrate treatment were subjected to CO_2_ flushing and pre-warmed in a water bath at 39°C. The bottles were sealed with a rubber and aluminum cap and placed in an incubator at 39°C. The rumen-fluid mixture was added (40 mL) to the bottles and incubated at 39°C, as described in detail by Blümmel and Orskov [21].

### *In vitro* gas production, samplings and chemical analysis

During incubation, gas production of all the treatment samples was recorded at 1, 2, 4, 6, 8, 12, 24, 48, 72, and 96 h. The cumulative gas produced during fermentation was fitted to the model of Ørskov and McDonald [22]. After inoculation, the pH of the ruminal fluid was determined at 4, 8, and 12 h, with gas production recorded at each time point. The rumen fluid samples were then divided into two parts. The first portion of 20 mL rumen fluid was collected in a plastic bottle, added with 2 mL of 1 mol L^−1^ sulfuric acid (H_2_SO_4_) and centrifuged at 16,000×g for 15 min. The supernatant was removed and stored at −20°C for later NH_3_-N analysis using the micro-Kjeldahl method [15] and volatile fatty acid (VFA) analysis according to Samuel et al [23]. The second portion was subjected to a total direct counting approach of the protozoal population [24]. After incubation, the *in vitro* DM degradability (g/kg) was determined at 12 and 24 h according to Van Soest and Robertson [25]. CH_4_ production was measured using a gas chromatograph (GC) machine (GC2014; Shimadzu Co., Ltd., Kyoto, Japan); a volume of 0.3 mL of the gas was kept in a 10 mL bottle, followed by injecting the gas using a GC. Based on the equation: methane production = (peak area/the slope of the standard methane graph)/gas volume (mL).

### Statistical analyses

All experimental data were analyzed by the general linear model by using the procedure of SAS software [26] as a 2×4×2 factorial arrangement in a CRD. The statistical model included R:C ratio, AMP levels, TUP levels, R:C ratio×AMP level interactions, R:C ratio×TUP level interactions, AMP level×TUP level interactions, and R:C ratio×AMP level×TUP level interactions. Differences among statistical treatment parameters were taken as significant at p<0.05 and p<0.01.

## RESULTS AND DISCUSSION

### Chemical composition of experimental feeds

Concentrate, rice straw, AMP, and TUP chemical compositions are listed in Table 1. The crude protein of the concentrate and rice straw were 14.2 and 2.2%. The AMP had 5.6% DM, 7.2% ash, 23.6% CP, 64.4% NDF, 54.4% ADF, 2.0% total phenolics, 3.6% total flavonoids, and 1.8% tannins. Moreover, TUP contained 88.3% DM, 6.4% ash, 4.5% CP, 40.4% NDF, 26.1% ADF, 8.3% total phenolics, 18.0% total flavonoids, and 16.6% tannins, respectively. In the other hands, analyzed the chemical composition of CP in *Azolla microphylla* and found it to be at a concentration of 31.3% [7]. However, Hasan and Chakrabarti [4] stated that the CP content of *Azolla* was typically between 19.0% and 30.0% under circumstances for optimal growth.

### Gas production kinetics and feed degradability

The gas production kinetics and *in vitro* true DM degradability are presented in Table 2. Gas production kinetics included gas production from the insoluble fraction (b), gas production rate constant for the insoluble fraction (c), the potential extent of gas production (a + b), and cumulative gas production at 96 h were affected by R:C and numerically increased with AMP and TUP supplementation (p<0.05) but no interactions were observed. Gas production kinetics increased with increasing ratios of concentrate and AMP supplementation, whereas TUP supplementation reduced gas production. *In vitro* DM degradability at both 12 and 24 h after incubation remarkably increased (p<0.05) with R:C ratio, AMP, and TUP supplementation, with the lowest found in the non-supplementation group. However, no interaction was found (p>0.05).

The degradabilities improved with an increase in the ratio of concentrate and increased with the addition of AMP supplementation. The fermentation process that occurs with the feed substrates can have a positive effect on the total amount of rumen gas produced. A substantial relationship has been found between the variety of roughages utilized and the concentration of carbohydrates present in feeds and rations, with the potential to greatly influence the production of gases. Previous research by Viennasay et al [27] found that feeds and plants containing phytonutrients such as CT and saponins had antibacterial capabilities against a variety of microorganisms including rumen bacteria, protozoa, and fungi. Nevertheless, Phesatcha et al [28] stated that gas production kinetics were improved by Mitragyna leaf powder, possibly because it enhanced the microbial population (particularly bacteria population) and increased substrate breakdown, thereby enhancing gas production kinetics. However, feed digestibility was unaffected by or enhanced by the addition of herbal extracts containing tannins and saponins. Similarly, no significant difference (p>0.05) was recorded in the digestibility of nutrients with the addition of 10 and 20 g/kg turmeric DM [29].

### Rumen fermentation

Results in Table 3 show the impacts of factors on pH, ammonia-nitrogen concentration, and protozoal population. The ruminal pH was not affected (p>0.05) by R:C, AMP, and TUP supplementation, while the rumen ammonia-nitrogen concentration reduced (p<0.05) with increasing levels of R:C, AMP, and TUP supplementation. No interaction was found between R:C, AMP, and TUP while the protozoal population decreased (p<0.05) with R:C ratio, AMP, and TUP supplementation but with no interactive results. Our results showed that the pH of the rumen was within the typical range of 6.6 to 6.8 which is considered normal for the ecology of the rumen as well as for the effectiveness of the fermentation process. Similarly, Van Soest [30] concluded that the most suitable pH range for microbial activity was between 6.2 and 7.2. AMP and TUP supplementation decreased the ammonia-nitrogen concentration. The protein-CT binding interaction may be responsible for the drop in ruminal NH_3_-N concentration observed when supplemented with TUP. Supplementation with *Azolla* improved the fermentation process in the rumen by supplying the microorganisms with more nutrients, which increased their capacity to degrade feed. Ammonia production from feed protein breakdown due to reduced deamination caused the rumen pH to drop significantly after adding 1% DM curcuma powder [31], while AMP and TUP supplementation decreased NH_3_-N content. The tannin-protein complex played a role in the proteolysis process, which inhibited the breakdown of proteins and reduced the concentration of NH_3_-N. Similarly, Wanapat et al [32] and Patra and Saxena [33] who demonstrated that CT possessed dietary benefits from the production of a tannin-protein complex which reduced the availability of ruminal breakdown feed protein, and the production of NH_3_-N. The relationship between protein and tannin in temulawak showed potential for decreasing NH_3_-N concentration [34]. According to Sulistyowati et al [35] suggested that the active compounds found in curcuma, which have been shown to enhance the reduction of ammonia, contributed to a decrease in the number of deaminator bacteria. The reduction in ammonia nitrogen was attributed to inhibition of substrate protein degradation by microorganisms. The decreasing effect that turmeric has on ammonia nitrogen could be advantageous in terms of enhancing the use of dietary proteins in the rumen. Furthermore, the protozoal population also greatly decreased. Methanogens that are adherent on the surface of protozoa are negatively impacted by the presence of tannins and flavonoids, and this has an adverse effect on the population of protozoa. A correlation was found between the phytochemicals in turmeric, which have been associated with antibacterial properties, and the reduced effect that turmeric had on the population of microorganisms. Furthermore, Tyagi et al [14] reported that curcumin possessed powerful antibacterial effects against both gram-positive and gram-negative bacteria, while tannins, flavonoids, saponins, and oxalates have all been shown to have antibacterial properties [36,37]. Hence, the observed fermentation and microbial population decreases were directly associated with the effect of curcumin and other plant secondary metabolites contained in turmeric. The secondary metabolites found in plants possess a wide variety of antibacterial actions. These include disruption of the cell membrane, inhibition of enzymes, deprivation of substrates, and prevention of bacterial colonization [38]. Similarly, Phesatcha et al [28] showed that supplementation of Mitragyna leaves resulted in a decrease in the population of ruminal methanogens as well as the production of methane. Protozoa host methanogen archaea and also feed on the bacteria responsible for feed fermentation. Reducing protozoa can therefore improve the digestibility of DM, organic matter, and VFA, which are then turned into energy in the livers of ruminants.

### Rumen volatile fatty acids concentration and methane production

Table 4 presents the details of total VFAs (C_2_, C_3_, C_4_, and the C_2_:C_3_ ratio) and rumen CH_4_ production. The R:C ratio increased with the addition of AMP and TUP, and the levels of propionate (C_3_) and total VFA also increased (p<0.05), while acetic acid (C_2_) and the ratio of C_2_ to C_3_ decreased (p<0.05). The addition of TUP resulted in a decrease in the production of VFA, while methane production was reduced by increasing the R:C ratio and TUP supplementation (p<0.05) at all times of incubation. However, no interaction was found between the R:C ratio, AMP, and TUP regarding VFA or methane production. In this study, supplementation with AMP and TUP resulted in a significant improvement in the production of propionate, as well as total VFA production. Accordingly, Ahmed et al [39] reported that the utilization of plant-based bioactive supplements resulted in a significant enhancement of rumen fermentation, particularly regarding the production of total VFA and propionate under *in vitro* conditions. The microbes used the hydrogen created during rumen fermentation to increase propionate production, which in turn improved total VFA production [3]. For ruminants, VFA are the primary source of energy for minerals and the liver is responsible for converting VFA into glucose [40]. When the value of VFA is high, this suggests that the process of turning feed into energy in the rumen continues to function correctly. Accordingly, Sulistyowati et al [35] reported that the most effective utilization of VFAs occurred when the dose of curcuma powder was 0.25% of DM. This resulted in a reduction in protozoa that ingest fiber-digesting bacteria and an increase in VFA values. The availability of phytonutrients including flavonoids and tannins also contributed to the decrease in CH_4_ production induced by AMP and TUP supplementation. Specifically, as the supplemental level increased, CH_4_ production decreased. Furthermore, Jayanegara et al [41] reported that tannins in proportions of more than 5% are not advised because they suppress the digestibility and performance of livestock. Including a tannin level of 2% to 5% in the diet had the most effective impact in decreasing methane gas production. By contrast, Al-Hadeethi et al [29] reported that *in vitro* studies had no significant impact on rumen methane production when turmeric was added at 10, 20, and 30 g/kg DM in the diet. Essentially, accordingly to Hristov et al [42] reported that the inclusion of plant-based feeds containing phytonutrients showed the potential to decrease the protozoal population and methane production, while simultaneously enhancing the concentration of VFAs in the rumen. In addition, Beyzi [43] stated that essential oils from plants effect the ecology of the rumen by influencing the protozoa and bacterial cell membranes, as well as reducing hydrogen content and inhibiting methanogenesis. Thus, the essential oils present in turmeric powder may contribute to the decrease in methane production.

## CONCLUSION

Our results indicated that supplementation with 9% AMP and 2% TUP greatly enhanced rumen nutrient degradability and fermentation end-products, especially propionate production and decreased methane production. AMP and TUP showed potential as a supplement to improve rumen fermentation. However, further *in vivo* research should be conducted to confirm our results, especially in beef cattle.

## Figures and Tables

**Table 1:** Chemical composition of concentrate, rice straw, *Azolla* powder, and turmeric powder used in the experiment

Items	Concentrate	Rice straw	AMP	TUP
Feed ingredients (% of DM)				
Cassava chip	60.0			
Coconut meal	15.0			
Rice bran	10.0			
Palm meal	10.0			
Molasses	2.0			
Urea	1.5			
Sulfur	0.5			
Mineral mixed	0.5			
Salt	0.5			
Chemical composition				
Dry matter (%)	92.5	93.0	5.6	88.3
Organic matter (% DM)	92.6	91.5	92.8	93.6
Ash (% DM)	7.4	8.5	7.2	6.4
Crude protein (% DM)	14.2	2.2	23.6	4.5
Neutral detergent fiber (% DM)	14.6	75.5	64.4	40.4
Acid detergent fiber (% DM)	18.7	47.4	54.4	26.1
Total phenolics (% DM)			2.0	8.4
Total flavonoids (% DM)			3.6	18.0
Tannins (% DM)			1.8	16.6

AMP, *Azolla* (*Azolla microphylla*) powder; TUP, turmeric (*Curcuma longa*) powder; DM, dry matter.

**Table 2:** Using *Azolla* (*Azolla microphylla*) leaf meal and phytonutrient powder on gas kinetics and nutrient degradability

Treatment	R:C	AMP	TUP	Gas kenetics^1)^				Cumulative gas (mL) at 96 h	*In vitro* DM degradability (%)	
	
				a	b	c	a+b		12 h	24 h
T1	60:40	0	0	1.6	50.8	0.04	52.4	51.5	50.1	52.8
T2			2	2.0	54.6	0.03	56.6	46.4	50.6	59.8
T3		3	0	3.6	56.4	0.03	60.0	33.2	63.8	58.3
T4			2	3.3	76.2	0.03	79.5	59.7	65.0	60.5
T5		6	0	2.9	75.5	0.01	78.4	64.4	53.7	67.4
T6			2	5.2	88.9	0.01	94.1	75.2	62.0	71.2
T7		9	0	2.0	74.2	0.01	76.2	81.1	50.3	63.8
T8			2	1.4	76.2	0.01	77.6	71.5	54.7	75.1
T9	40:60	0	0	1.2	60.5	0.02	61.7	60.4	58.6	55.5
T10			2	1.4	65.4	0.03	66.8	84.6	62.3	77.6
T11		3	0	0.6	76.6	0.03	77.2	89.8	64.1	63.9
T12			2	1.1	83.2	0.03	84.3	77.5	68.2	87.0
T13		6	0	2.1	68.5	0.02	70.6	89.0	70.1	81.3
T14			2	2.8	73.8	0.02	76.6	49.2	72.5	86.7
T15		9	0	2.4	80.3	0.04	82.7	94.8	67.4	80.0
T16			2	1.3	86.6	0.02	88.0	83.8	69.3	83.7
SEM				1.71	1.35	0.007	0.48	0.48	1.02	1.25
R:C				**	**	*	**	**	*	*
AMP				*	*	*	*	*	*	*
TUP				ns	*	ns	*	*	ns	*
R:C×AMP				ns	ns	*	*	ns	ns	ns
R:C×TUP				**	ns	*	ns	**	*	*
AMP×TUP				ns	ns	ns	ns	ns	ns	ns
R:C×AMP×TUP				ns	ns	ns	ns	ns	ns	ns

R:C, roughage-to-concentrate ratio; AMP, *Azolla* (*Azolla microphylla*) powder; TUP, turmeric (*Curcuma longa*) powder; DM, dry matter; SEM, standard error of the mean; ns, not significant

1) Gas production, a, the gas production from the immediately soluble fraction; b, the gas production from the insoluble fraction; c, the gas production rate constant for the insoluble fraction (b); a+b, the gas potential extent of gas production.

* p<0.05,

** p<0.01.

**Table 3:** Using *Azolla* (*Azolla microphylla*) leaf meal and phytonutrient powder on ruminal pH, ammonia-nitrogen concentration and protozoal population

Treatment	R:C	AMP	TUP	pH	NH_3_-N (mg/dL)	Protozoa (×10^5^ cells/mL)
T1	60:40	0	0	6.89	18.6	5.0
T2			2	6.86	17.1	7.5
T3		3	0	6.83	17.5	6.3
T4			2	6.83	16.9	5.5
T5		6	0	6.85	16.4	6.0
T6			2	6.82	16.8	3.6
T7		9	0	6.88	15.7	5.5
T8			2	6.83	15.1	3.7
T9	40:60	0	0	6.72	22.4	11.5
T10			2	6.75	21.8	3.5
T11		3	0	6.78	21.1	4.0
T12			2	6.77	20.3	6.1
T13		6	0	6.82	19.5	8.0
T14			2	6.79	18.1	4.3
T15		9	0	6.74	18.8	7.5
T16			2	6.71	17.2	3.5
SEM				0.03	1.08	1.30
R:C				ns	*	*
AMP				ns	**	**
TUP				ns	*	**
R:C×AMP				ns	ns	ns
R:C×TUP				ns	*	*
AMP×TUP				ns	**	ns
R:C×AMP×TUP				ns	ns	ns

R:C, roughage-to-concentrate ratio; AMP, *Azolla* (*Azolla microphylla*) powder; TUP, turmeric (*Curcuma longa*) powder; NH_3_-N, ammonia-nitrogen; SEM, standard error of the mean; ns, not significant.

* p<0.05,

** p<0.01.

**Table 4:** Using *Azolla* (*Azolla microphylla*) leaf meal and phytonutrient powder on volatile fatty acids and methane production

Treatment	R:C	AMP	TUP	VFA (mol/100 mL)				Total VFA (mmol/L)	Methane production (mL/0.5 g DM)	
	
				C_2_	C_3_	C_4_	C_2_:C_3_		12 h	24 h
T1	60:40	0	0	65.8	15.0	8.4	4.3	42.4	6.7	11.9
T2			2	64.0	16.5	10.5	4.0	51.5	6.3	10.1
T3		3	0	72.2	18.6	10.8	3.8	57.2	5.8	11.9
T4			2	73.5	16.8	11.7	4.3	68.8	6.0	10.2
T5		6	0	76.1	16.4	10.9	4.6	62.2	5.2	9.9
T6			2	75.2	20.9	10.8	4.0	65.7	4.6	8.5
T7		9	0	78.6	18.7	8.7	4.2	73.6	5.7	9.1
T8			2	71.8	20.8	12.5	3.4	75.4	4.5	8.2
T9	40:60	0	0	58.1	14.7	10.1	4.0	65.2	4.8	7.5
T10			2	53.9	17.9	9.0	3.0	68.7	3.8	6.8
T11		3	0	63.1	19.1	14.1	3.3	77.1	2.7	3.4
T12			2	67.2	20.4	17.5	3.2	79.3	2.4	4.0
T13		6	0	66.8	23.9	13.4	2.8	84.2	4.6	8.8
T14			2	56.2	26.7	17.8	2.1	80.4	3.8	7.8
T15		9	0	72.3	25.8	12.0	2.8	88.7	4.0	7.6
T16			2	66.9	28.4	15.2	2.3	82.9	3.1	5.3
SEM				1.46	2.03	1.52	0.45	2.16	0.25	0.89
R:C				*	**	*	*	**	**	**
AMP				ns	**	ns	ns	**	*	**
TUP				*	**	ns	*	**	**	**
R:C×AMP				ns	ns	ns	ns	ns	ns	*
R:C×TUP				ns	*	ns	ns	*	ns	*
AMP×TUP				ns	ns	ns	ns	ns	ns	ns
R:C×AMP×TUP				ns	*	ns	ns	ns	ns	ns

R:C, roughage-to-concentrate ratio; AMP, *Azolla* (*Azolla microphylla*) powder; TUP, turmeric (*Curcuma longa*) powder; VFA, volatile fatty acids; DM, dry matter; C_2_, acetate; C_3_, propionate; C_4_, butyrate; C_2_:C_3_, acetate to propionate ratio; SEM, standard error of the mean; ns, not significant.

* p<0.05,

** p<0.01.
